# RETRACTION: PEDF and 34‐mer Peptide Inhibit Cardiac Microvascular Endothelial Cell Ferroptosis via Nrf2/HO‐1 Signalling in Myocardial Ischemia‐Reperfusion Injury

**DOI:** 10.1111/jcmm.71210

**Published:** 2026-05-21

## Abstract

**RETRACTION**: P. Lu, Y. Qi, X. Li, C. Zhang, Z. Chen, Z. Shen, J. Liang, H. Zhang, and Y. Yuan, “PEDF and 34‐mer Peptide Inhibit Cardiac Microvascular Endothelial Cell Ferroptosis via Nrf2/HO‐1 Signalling in Myocardial Ischemia‐Reperfusion Injury,” *Journal of Cellular and Molecular Medicine* 28, no. 14 (2024): e18558. https://doi.org/10.1111/jcmm.18558.

The above article, published online on 24 July 2024 in Wiley Online Library (wileyonlinelibrary.com), has been retracted by agreement between the authors; the journal Editor‐in‐Chief, Stefan Constantinescu; and John Wiley & Sons Ltd. A third party reported on PubPeer [1] that the majority of images in Figure 4D contained overlapping sections and that the HR+Fe‐citrate(III)+PEDF images had been duplicated between Figures 4D and 5A. All affected images represent different experimental conditions.

The authors responded to an inquiry by the publisher. However, the corrected data supplied by the authors could not be verified as having been derived from the original experiments.

The retraction has been agreed to because the evidence of image overlap and duplication fundamentally compromises the editors’ confidence in the results presented.
